# Assisted reproductive treatments in Urban Asia: A cross-sectional analysis of awareness, knowledge and attitude on fertility treatments in Hong Kong

**DOI:** 10.1177/17455057261460283

**Published:** 2026-06-08

**Authors:** Selina Tsz Ching Lee, Keith Siu Kay Yeung

**Affiliations:** 1 Department of Obstetrics and Gynaecology, 10051 Princess Margaret Hospital, Hong Kong, China; 2 Department of Anaesthesiology & Operating Theatre Services, 156807 Queen Elizabeth Hospital, Hong Kong, China

**Keywords:** assisted reproductive techniques, attitude, awareness, infertility, knowledge

## Abstract

**Background:**

Infertility affects 1 in 6 couples, with significant variations in awareness and acceptance of fertility treatments worldwide.

**Objectives:**

This study aimed to evaluate the awareness, knowledge and attitude on fertility treatments among the reproductive-age population in Hong Kong.

**Design:**

This was a cross-sectional questionnaire study.

**Methods:**

The questionnaires were distributed to individuals at the public area of the Obstetrics and Gynaecology clinic.

**Results:**

Of the 506 subjects recruited, 497 (98.2%) responded. While 65% demonstrated awareness of reproductive treatments, only half understood infertility’s definition and prevalence. Univariate logistic regression identified older age (OR 1.08, p<0.001) and better education level (OR 1.87, p=0.004) as significant predictors of awareness. Acceptance was reported by 70% of respondents, particularly among those with better knowledge and no religious beliefs.

**Conclusion:**

The findings underscore gaps in public awareness and knowledge on fertility treatments. It highlights the need for targeted education campaigns and policy enhancements to improve acceptability and accessibility.

## Introduction

Global fertility rates have plummeted from 5.3% in 1963 to 2.3% in 2022,^1^ with Hong Kong recording one of the world’s lowest total fertility rates at 0.751 in 2023.^2^ The birth rate in Hong Kong had also declined from 0.7% in 2019 to 0.44% in 2023.^2^ According to the latest territory-wide survey conducted by the Family Planning Association of Hong Kong in 2022, the number of childless women more than doubled to 43.2% compared with five years earlier, whereas those with one child and two children fell to approximately one quarter.^3^ The average parity among reproductive-age women has dropped to a new record low of 0.9, from 1.3 in 2017.^3^ While a number of couples may decide to be childless due to complex socio-economic factors,^4^ many couples struggle with infertility.

Modern societies worldwide are confronting a demographic shift characterized by two interrelated phenomena: rising voluntary childlessness and increasing infertility rates. Across high- and middle-income nations, surveys consistently show growing proportions of adults opting against parenthood. According to the territory-wide survey conducted by the Family Planning Association of Hong Kong in 2022, the percentage of those preferring to be childless increased to 22.7% for women and 20.1% for men in 2022, from approximately 8% for both sexes in 2017.^3^ The top reasons cited by respondents for not having children were fear of child-rearing responsibilities, society being unsuitable for children’s development and heavy financial burdens. Simultaneously, infertility, affecting 1 in 6 couples globally,^5^ has grown alongside voluntary childlessness. There is a rising trend in infertility due to a global rise in the age of marriage and first conception.^6^ The growing demand for assisted reproductive treatments (ART) reflects these dual trends, with ART cycles growing by 5-10% per annum over the past few years.^7^ According to statistics from the Council on Human Reproductive Technology, the number of total reproductive treatment cycles in Hong Kong has increased from 7511 in 2020 to 8494 in 2023, reflecting an increasing demand for assisted reproduction.^8^ These demographic challenges carry important public health and socioeconomic implications. The decline in birth rates impedes population growth. Together with population ageing, these factors hamper the overall productivity of society as well as the city’s economic growth. Therefore, policymakers globally, including those in Hong Kong, are seeking solutions to boost fertility rates and sustain workforce productivity and economic growth.

In an attempt to increase fertility rates, one of the strategies undertaken is to improve the utilisation of ART for those in need. Therefore, understanding the factors and barriers that determine access to care is critical. ART encompasses a range of medical procedures designed to address infertility by handling both eggs and sperms to facilitate conception. There are significant variations in awareness, knowledge, and attitude on ART across regions and cultural backgrounds. Different studies across the globe highlighted gaps in public fertility awareness and understanding of ART, even in high-income settings. For instance, a study in Australia showed that only a quarter of the respondents accurately understood their age-related fertility potential.^9^ Another recent study in the United Kingdom in 2024 showed that only 32% to 55% of respondents showed an understanding of in-vitro fertilization outcomes and 76% felt their education in fertility was inadequate.^10^ Women of minority groups demonstrated generally even poorer knowledge and lower awareness towards fertility treatments.^11^ Acceptance of ART is not uniform and is profoundly shaped by inter-contextual factors spanning cultural, socioeconomic, legal and ethical domains. Culturally, perceptions and acceptability of ART vary considerably across religious backgrounds.^12,13^ Socioeconomically, ART remains a costly intervention, with limited public funding in many regions like Hong Kong. This contrasts with countries such as Israel and parts of Europe where national health systems subsidize most of the ART expenditures.^14^ Such disparities in healthcare financing models also affect acceptance of ART. Legally and ethically, acceptance of ART is affected by local regulations determining which procedures are permitted, who can access them, and under what conditions, including preimplantation genetic testing, gamete donation, surrogacy, etc.^15^

This cross-sectional study aimed to evaluate the situation in Hong Kong, a high-income Asian society with diverse cultural backgrounds. We aimed to look into the awareness, knowledge, and attitude towards ART among the general public in Hong Kong. By identifying critical gaps in reproductive health literacy and barriers to ART utilization, this study sought to inform targeted public health strategies and clinical practices to address the low fertility rates through evidence-based interventions.

## Methods

Ethical approval for the study was obtained from the Central Institutional Review Board. (Ref no.: CIRB-2023-197-2, Date: 13th May 2024) Informed consent was obtained from all respondents.

This was a cross-sectional pilot questionnaire survey of the reproductive-age population aged 18-40 years. The recruitment period was from June to September 2024. Ethical approval for the study was obtained from the Central Institutional Review Board. (Ref no.: CIRB-2023-197-2, Date: 13th May 2024) All procedures were performed in compliance with relevant laws and institutional guidelines. Privacy rights of human subjects were observed. The questionnaires were distributed to individuals aged 18 to 40 years, accessible through the waiting area of the Obstetrics and Gynaecology (O&G) Clinic at Princess Margaret Hospital, Hong Kong. Participants attending the O&G clinics were thought to be more knowledgeable and more aware of ART, and served as a reference to those likely to receive the service in the region. Subjects who could not read Chinese or English were excluded from the study. The self-administered questionnaire included an information sheet with an explanation of the survey together with a quick response code directed to the electronic questionnaire. The participants were invited to complete the questionnaires with their mobile phones in the waiting area. Written informed consent for data collection and statements for protection of privacy were given in the information sheet as well as explanations by the research staff. To ensure confidentiality, the questionnaire was anonymous, and a non-recognizable code was generated for each respondent. The questionnaire consisted of 34 questions in four parts. The first part focused on background demographic information. The second and third parts focused on their knowledge and attitudes towards assisted reproductive services. The last part focused on their perceptions of public fertility services in Hong Kong. The questionnaire was available in both traditional Chinese and English.

### Statistical analysis

The study population was estimated from the total population of reproductive-aged individuals (18–40 years old) in Hong Kong (n= 1815000).^2^ Using Yamane’s simplified proportions formula for calculating the sample size with a margin of error of 0.05 and correcting for 25% nonresponse, a sample size of 499 was needed. Statistical analysis was performed via SPSS (version 27.0; SPSS, Chicago, IL). The demographic data are presented as mean+/-standard deviation and count (percentage) for continuous and categorical data respectively. Analyses of the factors associated with awareness of, and acceptance of fertility treatments were calculated with the chi-square test and Mann‒Whitney U test for categorical and continuous variables, respectively. A p-value of less than 0.05 was considered statistically significant. The study was reported according to the STROBE reporting guidelines.^16^

## Results

### Demographics

A total of 506 questionnaires were distributed, and 497 responses were received, with a response rate of 98.2%. The baseline demographic data of the respondents are shown in Table 1. A total of 34.6% (172/497) of the respondents reported that they had encountered difficulty in conceiving before, and 13.9% (69/497) had received fertility treatments in the past.

**Table 1.** Demographic data of the respondents.

**Age**	32 +/- 4.87
**Ethnicity (n=497)**	
Chinese	469 (94.4%)
Nonchinese	28 (5.63%)
**Employment (n=497)**	
Full time	341 (68.6%)
Part-time	31 (6.24%)
Student	22 (4.43%)
Unemployed	103 (20.7%)
**Household income (HKD) (n=497)**	
Less than $10,000	41 (8.25%)
$10,000 - $29,999	188 (37.8%)
$30,000 - $50,000	116 (23.3%)
More than $50,000	152 (30.6%)
**Educational level (n=497)**	
Primary	5 (1.01%)
Secondary	160 (32.2%)
Tertiary or above	332 (66.8%)
**Religion (n=497)**	
Catholic	20 (4.02%)
Christian	59 (11.9%)
Buddhism	36 (7.24%)
Islam	15 (3.02%)
Others	7 (1.41%)
Nil	360 (72.4%)
**Marital status (n=497)**	
Single	87 (17.5%)
Cohabitation	31 (6.24%)
Married	371 (74.6%)
Divorced	7 (1.41%)
Widowed	1 (0.2%)
**Having child(ren) (n=497)**	
None	323 (65%)
One	127 (25.6%)
Two	37 (7.44%)
Three or more	10 (2.01%)
**Having plan for future childbearing (n=497)**	
Yes	382 (76.9%)
**Had encountered difficulty in conceiving (n=497)**	
Yes	172 (34.6%)
**Received fertility treatment before (n=497)**	
Yes - in vitro fertilisation	36 (7.24%)
Yes - intrauterine insemination	10 (2.01%)
Yes - ovulation induction	11 (2.21%)
Yes - others	12 (2.42%)
No	428 (86.1%)

Data are presented as the means +/- standard deviations or numbers (percentages).

### Awareness

Overall, 323 (65%) of the respondents had heard of ART, and 218/323 (67.5%) obtained information from friends and the internet. Those who had heard of ART were significantly older, with more of Chinese ethnicity, had higher monthly household income and had higher educational levels than those who had not (Table 2). Univariate logistic regression revealed that increasing age and educational level were significantly associated with awareness of ART (Table 3).

**Table 2.** Factors associated with awareness of assisted reproductive technology (ART).

​	Heard of ART (n=323)	Not heard of ART (n=174)	P Value
**Age**	32.6 +/- 5.27	29.9 +/- 6.36	<0.001*
**Ethnicity**			0.034*
Chinese	310 (96%)	159 (91.4%)	​
Non-Chinese	13 (4%)	15 (8.6%)	​
**Household income**			0.001*
HKD$30000 or more	192 (59.4%)	76 (43.7%)	​
Below HKD$30000	131 (40.6%)	98 (56.3%)	​
**Educational level**			<0.001*
Tertiary	234 (72.5%)	98 (56.3%)	​
Below tertiary	89 (27.6%)	76 (43.7%)	​
**Religion**			0.827
Yes	88 (27.2%)	49 (28.2%)	​
No	235 (72.6%)	125 (71.8%)	​
**Parity**			0.705
Nulliparity	208 (64.4%)	115 (66.1%)	​
Primi-/multiparity	115 (35.6%)	59 (33.9%)	​

The data are presented as the means +/- standard deviations or numbers (percentages).

*Statistically significant.

**Table 3.** Univariate logistic regression analysis of factors for predicting awareness of assisted reproductive technologies.

​	B	OR, 95% CI	P Value
Age	0.077	1.08, 1.04-1.12	<0.001*
Chinese ethnicity	0.581	1.79, 0.792-4.03	0.162
Household income	0.218	1.24, 0.817-1.89	0.309
Educational level	0.625	1.87, 1.22-2.87	0.004*

*Statistically significant.

### Knowledge

With respect to knowledge in infertility and assisted reproductive services, 44.5% (221/497) of the respondents correctly stated that infertility was defined as failure to conceive after one year of regular unprotected coitus. A total of 35.2% (175/497) were aware of the prevalence of infertility worldwide being around 1 in 6 couples, and just more than a quarter (138/497, 27.8%) were correct about the age of female fertility decline. 86.7% (431/497) correctly identified the different types of infertility treatment methods. Concerning the service provision in the public sector in Hong Kong, 54.7% (272/497) were aware that the correct age limit for government-subsidised fertility treatments was 40 years, whereas 29% (144/497) incorrectly believed that the age limit was 45 years or above.

### Attitude

**Figure 1.**
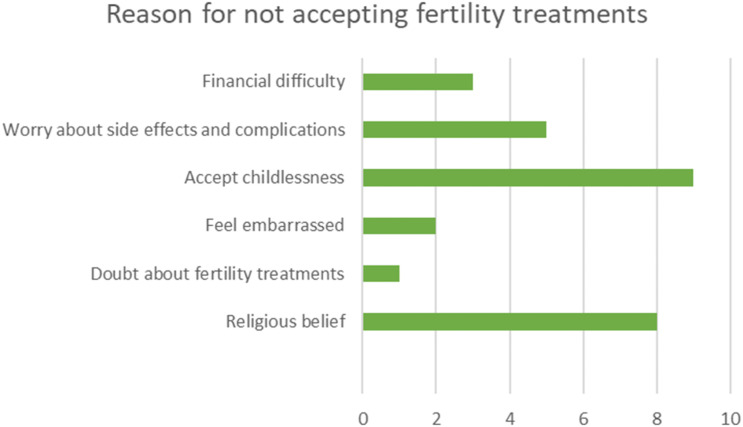
Reasons for declining fertility treatments.

**Figure 2.**
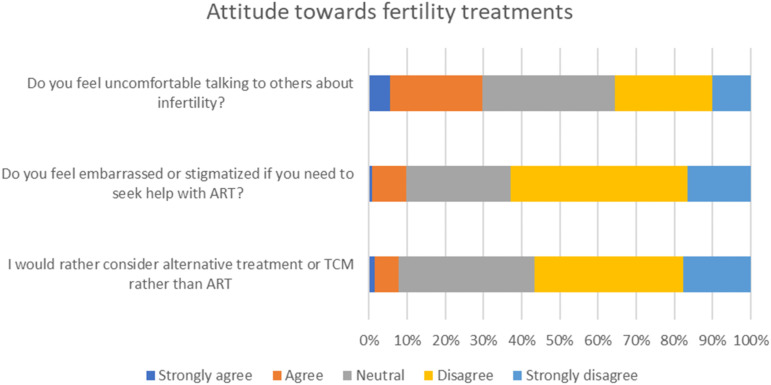
Attitude towards fertility treatments.

With respect to attitudes towards ART, 20.5% (102/497) strongly agreed, and 49.5% (246/497) agreed that they would accept fertility treatments if they failed natural conception. A total of 24.3% (121/497) were neutral about the issue, whereas 5.63% (28/497) disagreed. Those with no religious belief and those with better knowledge of the different fertility treatment methods had a significantly greater acceptance of fertility treatments, as shown in Table 4. Among those who did not accept fertility treatments (n=28), Figure 1 shows the main reasons for non-acceptance. 30% (148/497) of the respondents felt uncomfortable talking to others about infertility, whereas 9.9% (49/497) felt embarrassed or stigmatised for seeking help with fertility problems. 7.84% (39/497) would consider alternative treatments or traditional Chinese medicine rather than assisted reproductive technologies, as shown in Figure 2. 46.5% (231/497) would seek help from public hospitals or clinics for reproductive services if needed, while 32% (159/497) would resort to private hospitals or clinics. 77.3% (384/497) were in the opinion that fertility treatments were expensive. 37.6% (187/497) and 32.8% (163/497) were willing to spend less than HKD $10,000 (∼USD $1300) and between $10,000 and 30,000 (∼USD $3900) on fertility treatments, respectively. Only 12.9% (64/497) were willing to pay more than $50,000 (∼USD $6500).

**Table 4.** Factors associated with acceptance of assisted reproductive technologies.

​	Agree on fertility treatments (n=348)	Disagree on fertility treatments (n=28)	P Value
**Age**	32.2 +/- 5.73	30.3 +/- 5.93	0.078
**Ethnicity**			0.101
Chinese	334 (96%)	25 (89.3%)	​
Non-Chinese	14 (4%)	3 (10.75)	​
**Household income**			0.561
$30000 or more	206 (59.2%)	15 (53.6%)	​
Below $30000	142 (40.8%)	13 (46.4%)	​
**Educational level**			0.253
Tertiary	247 (71%)	17 (60.7%)	​
Below tertiary	101 (29%)	11 (39.3%)	​
**Religion**			0.043*
Yes	88 (25.3%)	12 (42.9%)	​
No	260 (74.7%)	16 (57.1%)	​
**Parity**			0.802
Nulliparity	228 (65.5%)	19 (67.9%)	​
Primi-/multiparity	120 (34.5%)	9 (32.1%)	​
**Knowledge on different methods of fertility treatments**			0.011*
Correct	307 (88.2%)	20 (71.4%)	​
Incorrect	41 (11.8%)	8 (28.6%)	​

The data are presented as the means +/- standard deviations or numbers (percentages).

*Statistically significant.

### Perception of assisted reproductive services in Hong Kong

A total of 60.6% (301/497) believed that public education on ART in Hong Kong was insufficient. 266/497 (53.5%) believed that there were not enough public fertility services in Hong Kong and 82.9% (412/497) agreed that the government should provide more funding for public fertility services. With increased government subsidies in ART, 65% (323/497) felt more encouraged to consider childbearing, and 68.4% (340/497) were more willing to consider fertility treatments if needed.

## Discussion

This study reviewed the latest situation in Hong Kong regarding the awareness, knowledge, and attitudes of the public towards assisted reproductive services as well as their perceptions of public fertility service provision. Our study demonstrated that awareness and knowledge of the public in Hong Kong regarding ART remained weak. Acceptance rates were strongly tied to knowledge levels. Most participants also advocated for increased government funding. No study had recently investigated the knowledge, attitude and perception of ART in Hong Kong nor the Asian population, which are key elements in gauging the effectiveness of the government funded programme.

Our study demonstrated several important findings. First, there was a rather low level of awareness of ART among our respondents, with only 65% of them having ever heard of assisted reproduction, which was much lower than a previous study in Europe.^17^ The disparity is striking given Hong Kong’s status as a high-income, highly developed international city with world-class healthcare infrastructure. This gap suggests that awareness is not merely determined by economic development but also cultural and social factors. Culturally, infertility remains a sensitive topic in Hong Kong, influenced by the traditional Chinese culture. It is a deep-rooted culture that infertility belittles an individual and it showed that even in an international modern city such as Hong Kong, there could still be a stigmatising and judgemental culture towards infertility and assisted reproduction. Infertility can carry stigma, perceived as a personal failing or a source of shame, which discourages open discussion and limits the diffusion of information about treatment options. This is echoed by our finding that nearly 30% of respondents felt uncomfortable talking to others about infertility. Those who were older and had higher educational levels had better awareness than their younger and less educated counterparts did. This pattern is consistent with studies in other high-income settings, where fertility awareness is often concentrated among higher socioeconomic groups.^18^ Therefore, public education campaigns must be specifically designed to reach younger, less-educated populations who may not proactively seek out fertility information. Moreover, most of the respondents obtained information from the internet and friends, reflecting a shift away from traditional health education channels. It may pose risks as online sources vary widely in accuracy and quality. This highlights the need for targeted outreach to less-educated groups, who predominantly access health information through traditional media such as television and newspapers. This suggests that a multimodal public education strategy is necessary, combining digital outreach with traditional media campaigns to ensure equitable information dissemination.

Our study also revealed critical gaps in knowledge on fertility and ART. Notably over 50% misunderstood infertility’s clinical definition and prevalence, while almost three-quarters underestimated age-related fertility decline. This knowledge gap echoes the findings of previous similar studies in other countries.^19,20^ Misperceptions about age-related fertility decline can lead to delayed childbearing and deferred help-seeking until it is too late for effective intervention. More importantly, barely more than 50% were aware of the age limit for government-funded assisted reproductive services. It is not uncommon to see women aged over 40 years in infertility clinics, for whom no treatment could be offered under the local practice guidelines, partly due to a misconception in the age limit for subsidised service in the public sector. The knowledge gap may be attributed to several factors. First, there is absence of comprehensive fertility education in Hong Kong’s school curricula. Second, fertility issues remain sensitive topics under the traditional culture and are rarely discussed openly within families or social networks. Third, the predominance of private sector ART services may create an information asymmetry, where knowledge is concentrated among those who can afford to seek out private consultations. Our study has shown that better knowledge is associated with increased acceptance of ART. Therefore, it is crucial to provide better education and campaigns at the territory-wide level to improve knowledge about infertility and fertility services in order to improve the utilisation of ART services.

Regarding the acceptance of ART, approximately 70% of our respondents expressed positive attitudes towards ART, indicating a general openness to fertility treatment among the Hong Kong public. This is an encouraging finding that suggests a foundation upon which to build more comprehensive fertility services. Our analysis demonstrated that those with no religious belief and those with better knowledge of different fertility treatment methods had significantly greater acceptance of ART. Hong Kong is a society where traditional Chinese religions coexist with Christianity and other faiths. Ethical perspectives on ART vary considerably. Understanding these religious perspectives is essential for developing culturally sensitive fertility education. On the other hand, approximately 30% of the respondents expressed that they felt uncomfortable talking to others about infertility. It is a deep-rooted culture that infertility belittles an individual and it showed that even in an international modern city such as Hong Kong, there could still be a stigmatising and judgemental culture towards infertility and assisted reproduction, which would create barriers to seeking help for those in need. This stigma creates a vicious cycle: when infertility is not openly discussed, accurate information does not circulate, misconceptions persist, and those who need help may suffer in silence. Increased public education can de-stigmatise infertility issues and can promote a positive and open culture to empower women to pursue fertility treatments.

Finally, financial constraints should be addressed. Our study has shown that financial limitations are a major factor in deterring couples from childbearing. Regarding ART service provision in Hong Kong, there are 35 licensed treatment centres that provide assisted reproductive treatments at the time of writing, and of these, among which 14 provide in vitro fertilization (IVF) services.^8^ The service landscape is predominantly private-sector driven, with only three of these IVF centres operating in the public healthcare system.^8^ Eligible couples can receive partial subsidies from the public sector under the Hospital Authority for up to three IVF cycles.^21^ Currently, the waiting time for couples receiving subfertility assessment in the public sector is approximately 7.5-12 months after referral. The waiting time for couples in need of IVF treatment is approximately four to seven months after the first consultation.^22^ The long waiting time for these services in public hospitals and the costs for private IVF often deters infertile couples from seeking treatment. An increase in the public fertility service quota and funding was generally welcomed by the public. Out-of-pocket IVF treatments in Hong Kong often exceeded HKD $100,000 (∼USD $13000), while the average monthly household income is around HKD $30000 (∼USD $3900).^23^ More than 60% of the respondents were only willing to pay less than HKD $30000 on fertility treatments. More than half of the respondents expressed a greater incentive to consider childbearing and fertility treatments with increased funding, underscoring the potential impact of policy intervention. Therefore, the government should consider further increasing subsidies for assisted reproductive services to promote service uptake and, ultimately, fertility and birth rates.

### Application of results in clinical practice and health policy

In clinical practice, fertility counselling during primary and gynaecological care can address the identified knowledge gaps, particularly regarding age-related decline and treatment eligibility. For health policy, strong public demand for increased funding and recognition of financial constraints as the primary barrier support expanding public ART quotas and funding. Territory-wide fertility education and destigmatization campaigns are needed.

Beyond clinical and policy reforms, nursing and allied health professionals occupy a frontline role in supporting individuals and couples navigating infertility. Community health nurses and midwives are uniquely positioned to initiate early conversations about fertility awareness during routine reproductive health visits, school health talks, and family planning consultations, where they can provide accurate psychoeducation on age-related fertility decline, the clinical definition of infertility, and available treatment pathways. Mental health professionals, including psychologists and social workers, should routinely screen for anxiety, depression, and social stigma-related distress among those undergoing or considering ART, as the emotional burden of infertility is often compounded by cultural shame and financial strain. Allied health professionals should also guide patients toward available financial and community resources, including subsidized public sector services, charitable funds, and peer support groups, thereby mitigating the disempowerment that often accompanies financial barriers. By embedding fertility support into routine nursing and allied health practice, Hong Kong can build a more accessible and multidisciplinary response to infertility—one that addresses not only clinical outcomes but also the psychological and social aspects of the fertility journey.

### Limitations

Our study was limited by its small sample size, and it included only subjects from a single unit. There was a potential selection bias by convenience sampling of subjects in the waiting areas of the O&G clinics, as those who are patients in our clinic may have underlying gynaecological conditions that might have prompted higher awareness on fertility issues. However, even this group of patients showed a lower awareness in ART than that shown by studies of the unselected general population abroad.^13^ Additionally, self-selection bias was also possible, as those with concerns about fertility issues may have been more likely to respond to the study. Nevertheless, this study was the first to review the current situation regarding public awareness, knowledge and attitudes towards assisted reproductive services in Hong Kong, providing insight regarding the issue in the Asia-Pacific region. This study provided useful information to inform improvements in public health policy and education to increase the acceptance and utilisation of assisted reproductive services in Hong Kong in steering towards a higher birth rate in the region. Future qualitative studies are needed to investigate specific cultural, religious or generational barriers unique to Hong Kong. Moreover, further cost-effectiveness studies on subsidizing ART in Hong Kong compared to the long-term societal costs of rising infertility are in need.

## Conclusion

Awareness and knowledge of the public in Hong Kong regarding ART remained weak. Acceptance rates were strongly tied to knowledge levels. Most participants also advocated for increased government funding. Our findings emphasized the need for targeted public education and policy reforms to bridge knowledge gaps and align ART services with population needs in the Hong Kong population.

## Data Availability

The data will be available upon reasonable request to the corresponding author.*
